# HAP1 can sequester a subset of TBP in cytoplasmic inclusions via specific interaction with the conserved TBP_CORE_

**DOI:** 10.1186/1471-2199-8-76

**Published:** 2007-09-14

**Authors:** Justin R Prigge, Edward E Schmidt

**Affiliations:** 1 Veterinary Molecular Biology, Molecular Biosciences, Montana State University, 960 Technology Blvd. Bozeman, MT 59717, USA; 2 Center for Reproductive Biology, Washington State University, Pullman, WA 99164, USA

## Abstract

**Background:**

Huntington's disease, spinal and bulbar muscular atrophy, and spinocerebellar ataxia 17 (SCA17) are caused by expansions in the polyglutamine (polyQ) repeats in Huntingtin protein (Htt), androgen receptor protein (AR), and TATA-binding protein (TBP), respectively. Htt-associated protein 1 (HAP1), a component of neuronal cytoplasmic stigmoid bodies (STBs), can sequester polyQ-expanded Htt and AR in STBs, thereby antagonizing formation of the nuclear aggregates associated with apoptotic neuron loss and disease progression.

**Results:**

Clones of HAP1 were isolated from unbiased two-hybrid screens for proteins that interact with TBP. Domain mapping showed that regions between amino acids 157 and 261 and between amino acids 473 and 582 of mouse HAP1 both bind specifically to the conserved C-terminal TBP_CORE _domain, away from the TBP N-terminal polyQ region. When fluorescently tagged versions of HAP1 or TBP were expressed independently in COS-7, 293, or Neuro-2a cells, all TBP localized to the nucleus and all HAP1 assembled into cytoplasmic stigmoid-like bodies (STLBs). When co-expressed, a portion of the TBP was assembled into the HAP1 STLBs while the remainder was localized to the nucleus. Although the TBP N terminus, including the polyQ region, was unnecessary for TBP-HAP1 interaction, in mammalian cells, removal of the TBP Qrepeat reduced the proportion of TBP that assembled into STLBs, whereas expansion of the Qrepeat had no significant affect on TBP subcellular localization.

**Conclusion:**

HAP1 can sequester a subset of TBP protein away from the nucleus; extranuclear TBP sequestration is quantitatively influenced by the TBP polyQ repeat. These results suggest HAP1 could provide protection from SCA17 neuropathology.

## Background

The Huntingtin-associated protein, HAP1, was first identified as a protein that interacts with Huntingtin protein (Htt), the causative agent of Huntington's disease (HD) [1-5]. HD is a member of a family of diseases in which expanded polyglutamine (polyQ) repeat regions in an otherwise functionally diverse group of proteins results in neurodegenerative disorders [1,3,6]. This family includes dentatorubral-palidoluysian atrophy (DRPLA), caused by polyQ expansion in atrophin 1; [7,8], spinal and bulbar muscular atrophy (SBMA or Kennedy's disease), caused by polyQ expansion in the androgen receptor, AR; [9-11], and spinocerebellar ataxias 1–3, 6, 7, and 17 (SCAs), caused by polyQ expansions in various proteins [1,3].

In all of these diseases, expansion of gene sequences encoding the polyQ region of the individual protein results in the disease in later life [1,3]. All are associated with neuronal accumulation of nuclear aggregates containing the affected protein as well as other proteins [1,3]. Although some extranuclear protein aggregates can often also be found, the nuclear accumulations are implicated in inducing the cytopathic state that leads to apoptotic loss of neurons [1]. In the case of HD, expansion of the polyQ repeat beyond ~36 residues causes an amino- (N-) terminal fragment (encoded by exon 1 of the *htt *gene) to be cleaved and to translocate to the nucleus, where it becomes incorporated into neuronal nuclear aggregates [12-14]. Nuclear HD aggregates typically contain proteasome subunits, chaperones, and ubiquitin [1,3,15,16]. Aggregates also contain transcription factors, including the TATA-binding protein (TBP), cyclic AMP response element-binding protein (CBP), specificity factor 1 (SP1), p53, and others [17,18]. One of these, TBP, is a general transcription factor that functions in initiation by all three nuclear RNA polymerases [19]. In addition, TBP is, itself, a polyQ protein that, upon expansion of its polyQ repeat, forms intranuclear aggregates leading to the SCA17 neuropathology [20]. Although TBP is generally a low abundance protein in normal somatic cells [21,22], the nuclear aggregates in SCA17 accumulate to high levels [23], suggesting that the pathological state is associated with increased accumulation of nuclear TBP.

Although HAP1 interacts with Htt, it is unclear what role this might play in HD. Recent evidence suggests that HAP1 may protect against polyQ-expansion neuropathologies [24-27], a proposal known as the 'HAP1 protection hypothesis' [25]. Thus, although expanded polyQ Htt is abundant throughout the brain, including in the HAP1-expressing hypothalamic and limbic regions in HD patients [25,28,29], these areas do not accumulate Htt aggregates and do not exhibit HD pathology [30]. Moreover, evidence for a neuroprotective role of HAP1 in SBMA has been reported. Thus, HAP1 binds to AR and SBMA pathology is generally not seen in neurons expressing HAP1 [27]. Importantly, whereas transfection of HEp-2 cells with polyQ-expanded AR results in apoptosis, co-transfection of HAP1 abrogates this effect [27].

Two HAP1 isoforms have been characterized in mice, HAP1-A, a 598 amino acid protein, and HAP1-B, a 628 amino acid protein, which both arise from the same gene [31]. The difference between HAP1-A and HAP1-B stems from alternative splicing in the 3' region of the pre-mRNA and results in both proteins containing different C-terminal sequences beyond the 577 amino acid HAP1 common region [31-33]. It is not yet clear how HAP1-A and HAP1-B might differ functionally, although the predominant vesicle-associated subtype is HAP1-B [32].

Subcellular localization studies show that HAP1 is predominantly cytoplasmic [34]. HAP1 is a component of stigmoid bodies (STBs) [34], which are non-membrane-bound cytoplasmic inclusions found in the hypothalamus and limbic regions of the brain [35]. HAP1 expression induces formation of cytoplasmic inclusions resembling STBs, here termed stigmoid-like bodies (STLBs), in 293 human embryonic kidney carcinoma cells, HEp-2 human larynx carcinoma cells, and mouse neuronal NIE 115 cells [27,36], suggesting that HAP1-A directs assembly of similar cytoplasmic inclusions in neuronal and non-neuronal cell types.

In the study presented here, unbiased yeast two-hybrid screens identified HAP1 as a protein that binds to TBP. Like the HAP1-interacting Htt and AR proteins, TBP is the causative agent of a polyQ-expansion-dependent neuropathology (see above). Moreover, like for Htt and AR proteins, we found that binding of HAP1 to TBP occurred away from the TBP polyQ region. Instead, interaction domain mapping studies showed that HAP1 binds specifically to the conserved C-terminal domain of TBP (TBP_CORE_). By inducing formation of green fluorescent protein-tagged HAP1 STLBs in 293, COS-7, or Neuro-2A cells, we show that cytoplasmic STLBs sequester some TBP while still allowing accumulation of substantial levels of TBP in the nucleus. These data suggest HAP1 can specifically associate with TBP to prevent nuclear localization of excess TBP while not impeding normal nuclear TBP accumulation and function.

## Results

### Identification of HAP1 as a TBP interacting protein

**Figure 1:**
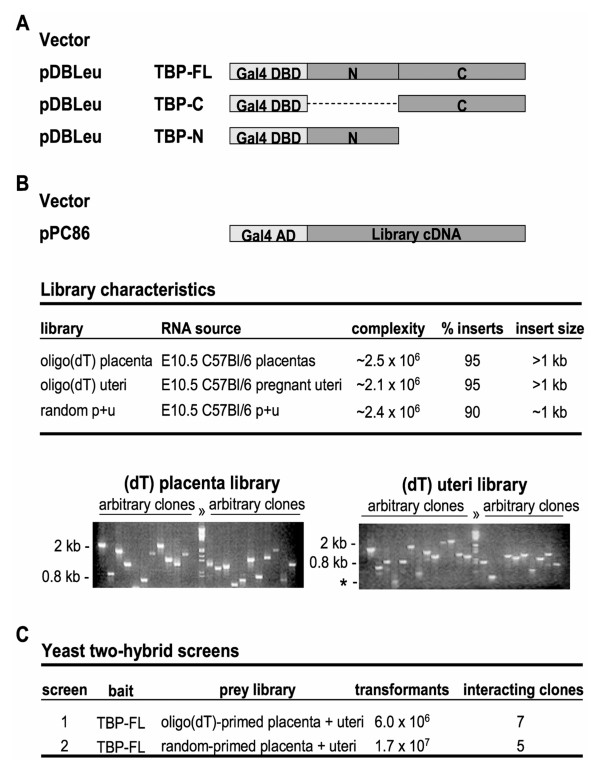
Yeast two-hybrid bait constructs, prey libraries, and screens. (A) Bait constructs. TBP-FL, TBP-C, and TBP-N were expressed from the pDBLeu plasmid, which fused the Gal4 DNA-binding domain (DBD) upstream of TBP. N and C designate the vertebrate-specific N terminus and the pan-eukaryotic TBP_CORE_, respectively. (B) Three prey libraries were constructed and inserted into pPC86, which fused prey cDNAs downstream of the Gal4 activation domain (AD). Characteristics of each library are indicated. Libraries were constructed from oligo(dt)-primed placental and pregnant uteri RNA or from random-primed placenta + pregnant uteri RNA (p+u). Below is shown PCR analysis of arbitrary clones from both of the oligo(dT)-primed libraries using a primer pair that spans the multiple cloning site of the vector. Lane "λ" contained *Hind *III/*Eco *RI-cut λ-phage DNA markers. Landmark band sizes are indicated at the left of each gel; the asterisk denotes the size of the PCR product arising from empty prey vector. (C) The results of the two yeast two-hybrid screens performed are shown. In both screens, TBP-FL was used as bait to screen either the oligo(dT)- or random-primed placenta + uteri prey libraries for interacting proteins. TBP-interacting prey library clones were subsequently identified by sequencing.

To identify proteins that interacted with TBP, we screened a mixture of oligo(dT)-primed placental and whole pregnant uteri cDNA libraries (50% of each library was used) (screen #1) or a random-primed library which also consisted of 50% placental and 50% whole pregnant mouse uteri cDNAs (screen #2). Both screens were performed using TBP-FL as the bait protein, which was expressed from the pDBLeu plasmid (Fig. 1). In screen #1, ~6 × 10^6 ^primary transformants (yeast containing both a bait and prey plasmid) were plated onto SC-L-W-H-U. From these plates, 82 colonies grew and were transferred to fresh SC-L-W-H-U plates and those clones that re-grew were further tested for activation of the LacZ reporter gene. Seven of the clones grew on SC-L-W-H + 50 mM 3-AT, SC-L-W-H + 75 mM 3-AT, SC-L-W-H-U, and also activated the LacZ reporter gene (data not shown). Inserts were sequenced to identify the interacting protein. Three clones encoded partial cDNAs of B'-related factor 1 (BRF1), a known TBP interacting protein [37]. A fourth clone encoded a nearly full-length HAP1-A protein, beginning at amino acid 7 (HAP1_7–598_). The remaining three clones encoded the following proteins: an unknown protein with similarity to human SR-rich proteins (accession number BAB30779) [38]; an unknown protein product containing similarity to chromosome segregation ATPases (accession number BAE22251); and Rho-associated coiled-coil forming kinase 1 (accession number NM_009071), a serine-threonine protein kinase.

**Figure 2:**
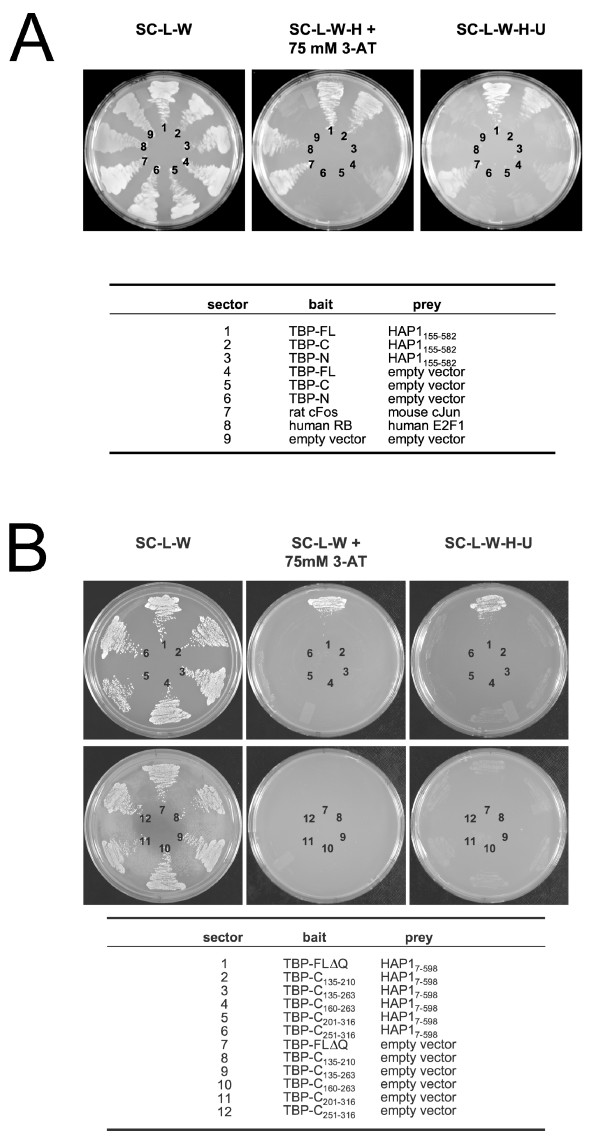
Protein interactions in the yeast two-hybrid system. Bait and prey plasmid combinations within individual yeast clones are indicated below. Sector designations correspond to those on plates. Growth of yeast on SC-L-W indicated that clones contained both the bait and prey plasmid. (A) Interaction with TBP-FL, TBP-N, and TBP-C. Sectors 1–3 show the interaction of HAP1-B_155–582 _with TBP-FL, TBP-C, or TBP-N, respectively. The interaction of HAP1 with TBP-FL and TBP-C, but not TBP-N resulted in growth under all conditions tested. Sectors 4–6 were auto-activation controls for TBP baits. Sectors 7–8 contained strong and weak positive interaction controls, respectively, that were supplied with the ProQuest system. The strong interaction control grew well under all conditions tested. The weak interaction control did not grow on SC-L-W-H-U, suggesting that this condition allowed growth of yeast containing only strong bait-prey interactions. Sector 9 was a negative auto-activation control showing that yeast bearing only the empty bait vector and an empty prey plasmid (no inserts) did not grow under conditions that selected for interacting bait-prey protein pairs. (B) Finer TBP deletions. Sectors 1–6 show the interaction of HAP1-A_7–598 _with TBP-ΔQ (sector 1) and truncated TBP-C clones (as indicated in table below; sectors 2–6). HAP1 interacted with TBP-ΔQ under all conditions tested (sector 1), but did not interact with any of the truncated TBP-C clones (sectors 2–6), suggesting that an intact TBP_CORE _is required for the HAP1-TBP interaction to occur. Sectors 7–12 were auto-activation controls for TBP baits, of which none were auto-active.

In screen #2, using the random-primed prey library, ~1.7 × 10^7 ^primary transformants were plated and tested as in screen #1. Out of 183 colonies that grew on SC-L-W-H-U, five yeast clones activated all three reporter genes (HIS3, ADE2, and LacZ), did not grow in the presence of an empty bait plasmid (no TBP insert; data not shown), and thus appeared to express a TBP-interacting prey protein. One of these clones encoded amino acids 155–582 of the HAP1-B (HAP1_155–582_) protein, with only the last five amino acids extending beyond the HAP1-common domain and into the HAP1-B-specific region. The other four clones were identical clones of cyclic AMP-dependent protein kinase regulatory protein α (accession number AAH03461) [39]. The interaction of the random-primed HAP1 clone with TBP-FL is shown in Figure 2A, sector 1. The HAP1-TBP-FL interaction and control protein interaction pairs are shown in Figure 2A, sectors 1 and 7–8, respectively.

### Interaction of HAP1 with TBP domains

To determine whether HAP1 interacted with the conserved TBP_CORE _or the vertebrate-specific TBP N terminus, yeast were co-transformed with either TBP-N or TBP-C bait plasmids and the HAP1_155–582 _prey plasmid. Growth of all clones on SC-L-W verified the presence of both the bait and prey plasmids (Fig. 2A, left panel). In the presence an empty prey plasmid, TBP-FL, TBP-C, and TBP-N did not grow under the protein interaction selection conditions (Fig. 2A, sectors 4–6). Similarly, yeast that contained either the empty bait or empty prey plasmids (negative controls) did not grow on plates that selected for interaction of bait and prey proteins (Fig. 2A, sector 9, panels 2, 3). The HAP1_155–582 _clone interacted with the TBP_CORE _(Fig. 2A, sector 2, panels 2, 3), but not with TBP N (Fig. 2A, sector 3, panels 2, 3).

The interaction of HAP1 with Htt or AR has been shown to occur away from the polyQ repeat region in each protein, but to be influenced by expansion of the polyQ repeat [12,27]. In two-component two hybrid screens, we found a strong interaction of HAP1 with either the TBP_CORE _alone (lacking the N-terminal polyQ region; Fig. 2A, sector 2, panels 2, 3) or with full length TBP protein in which the polyQ region had been replaced by two glycine residues (TBP-ΔQ; Fig. 2B). Thus, the TBP polyQ region showed no measurable contribution to the interaction of HAP1 with TBP in the yeast two-hybrid system.

All attempts at further truncating the TBP_CORE _region to define the interaction domain on TBP disrupted the TBP-HAP1 interaction (Fig. 2B, sectors 2–6), suggesting that the interaction might be dependent on the structural integrity of the entire TBP_CORE _region.

### Interaction of TBP with HAP1 in co-transfected cells

**Figure 3:**
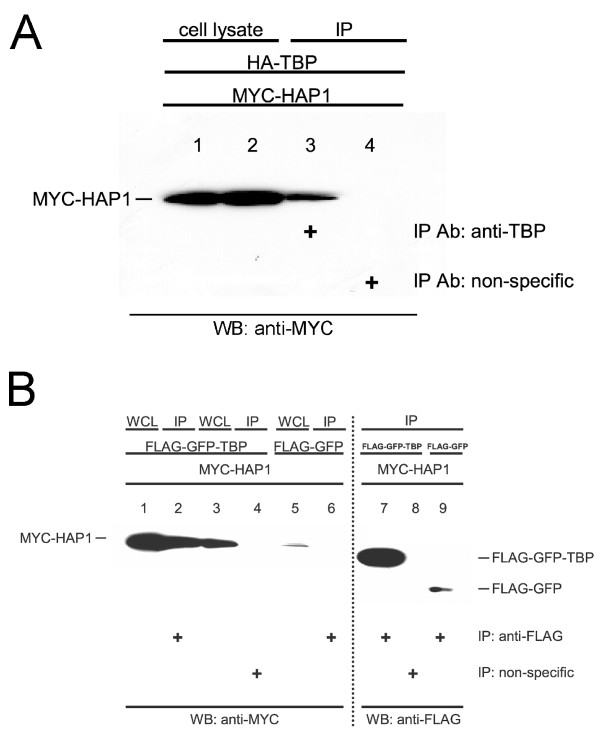
Co-immunoprecipitation assay. TBP/HAP1-B_155–582 _interactions in co-transfected cells. (A) 293 cells were co-transfected with pCMV-HA-TBP-FL and pCMV-MYC-HAP1. Whole cell lysates (WCL) (lanes 1–2, where lane 1 and lane 2 represent 4% of each WCL used for immunoprecipitation in lanes 3 and 4, respectively) or immunoprecipitated samples (lanes 3–4) were assayed using western blots with anti-MYC antibody. MYC-tagged HAP1 co-precipitated with TBP in the presence of the anti-TBP antibody (lane 3), but not with the non-specific antibody (lane 4). (B) COS-7 cells were co-transfected with pFLAG-CMV-GFP-TBP-FL (lanes 1–4, 7–8) or pFLAG-CMV-GFP (lanes 5–6, 9) and pCMV-MYC-HAP1-B_155–582 _(lanes 1–9). Whole cell lysates (lanes 1, 3, and 5, HAP1) or immunoprecipitated samples (lanes 2, 4, and 6, HAP1) were assayed using western blots and anti-MYC antibody. MYC-tagged HAP1 co-precipitated with FLAG-tagged-GFP-TBP in the presence of the anti-FLAG antibody (lane 2), but not with the non-specific antibody (lane 4). Myc-tagged HAP1 did not co-precipitate with FLAG-tagged-GFP in the presence of anti-FLAG antibody (lane 6). 30% of each immunoprecipitated sample was also blotted with anti-FLAG antibody (lanes 7–9) to verify the amount of FLAG-tagged protein that was captured in each co-precipitation assay (lanes 7–9 represent co-precipitated samples in lanes 2, 4, and 6, respectively).

In mammals, protein domains resembling the TBP_CORE _region are found only in TBP and its two homologues [40,41]. Similarly, the TBP-interacting domains in HAP1 are not found in other proteins [33]. Thus, the TBP-HAP1 interaction appeared to be a specific interaction between unique polypeptide domains. For verification, the interactions between TBP and HAP1 identified by two-hybrid screens were tested by co-immunoprecipitation in mammalian cell culture. Differently epitope-tagged versions of full-length TBP and HAP1_155–582 _were co-expressed in transiently transfected 293 cells. Results indicated that HAP1 co-precipitated with TBP when both proteins were co-expressed, but was not detected using a species-matched, nonspecific antibody (Fig. 3A). In a similar experiment, epitope-tagged HAP1_155–582 _co-precipitated with a GFP-TBP fusion protein (Fig. 3B), again verifying the interaction between HAP1 and TBP in mammalian cells.

### Mapping the TBP-interaction domain on HAP1

**Figure 4:**
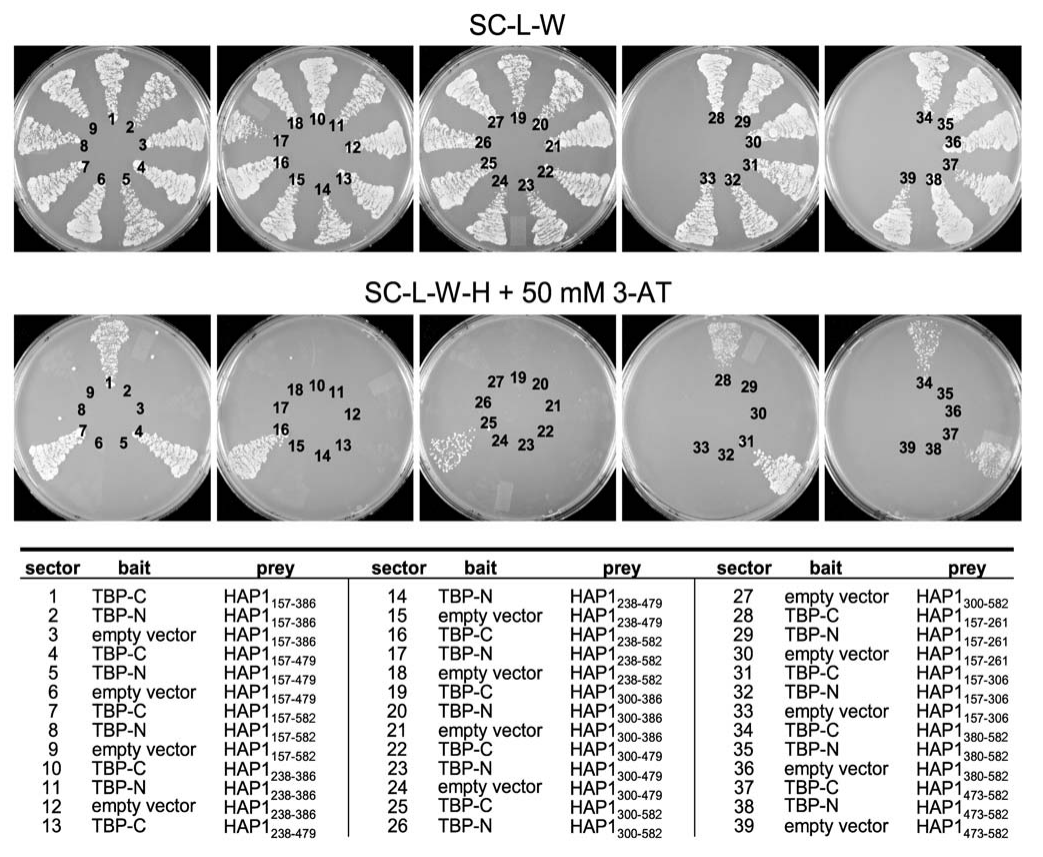
Identification of TBP/HAP1 interaction domains. The interaction of truncated HAP1 clones with TBP-C, TBP-N, and auto-activation controls are shown. Yeast containing the indicated bait and prey plasmids (listed below) were grown on SC-L-W (top panel), which selects for the presence of the bait and prey plasmids, and SC-L-W-H + 50 mM 3-AT (bottom panel), which selects for the interaction between bait and prey. All baits were expressed from the pDBLeu plasmid and all truncated HAP1 preys were expressed from the pPC86 plasmid. Sectors 3, 6, 9, 12, 15, 18, 21, 24, 27, 30, 33, 36, and 39 were controls confirming that truncated HAP1 clones were not auto-active. Sectors 2, 5, 8, 11, 14, 17, 20, 23, 26, 29, 32, 35, and 38 confirmed that none of the HAP1 clones interacted with TBP-N. Clones 10, 13, 19, and 22 did not grow on SC-L-W-H + 50 mM 3-AT, which suggested that these clones lacked the TBP interaction domain, whereas growth of clones 1, 4, 7, 16, 25, 28, 31, 34, and 37 on SC-L-W-H + 50 mM 3-AT suggested that these clones contained a TBP interaction domain. Three independent clones of all HAP1 truncations were tested and each gave the same result; one representative set is shown.

**Figure 5:**
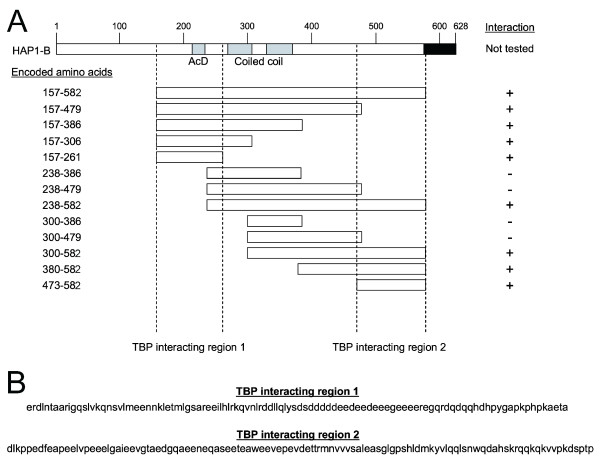
Deletion analysis of the TBP interacting regions on HAP1. (A) The full-length HAP1-B protein is represented at top, including the location of the known acidic domain (AcD) and the two coiled coil domains. HAP1 amino acids encoded by each cDNA clone in pPC86 are shown at left. The presence (+) or absence (-) of interaction with TBP-C is shown at right. Results suggested that the TBP interacting regions on HAP1 were between amino acids 157 and 261, and between amino acids 473 and 582. (B) Amino acid sequences of HAP1 TBP-interacting regions.

To identify the domain of HAP1 that interacted with TBP, we constructed truncated versions of HAP1 in the pDBLeu bait vector (Figs 4, 5). Each of the HAP1 truncated clones was tested for interaction with TBP-C and TBP-N in the two-hybrid system (Fig. 4, bottom panel). We found that all clones that began at amino acid 157 interacted with TBP-C (Fig. 4, sectors 1, 4, 7, 28, and 31; Fig 5), but not with TBP-N (Fig. 4, sectors 2, 5, 8, 29, and 32). The shortest N-terminal fragment of HAP1 that interacted with TBP encoded amino acids 157–261, which suggested that a TBP interacting region was between amino acids 157–261 of the HAP1 protein. The linear sequence of amino acids within TBP interacting region 1 is shown in Fig. 5. In addition, the lack of interaction between TBP-C and HAP1_238–479 _(Fig. 4, sector 13) suggested that this TBP interacting region on HAP1 required amino acids 157–237.

Some HAP1 clones that encoded amino acids outside TBP interacting region 1 also interacted with TBP-C (Fig. 4, sectors 16, 25, 34, and 37) but not with TBP-N (Fig. 4, sectors 17, 26, 35, and 38). The shortest C-terminal HAP1 clone that interacted with TBP-C encoded amino acids 473–582, which suggested that a second TBP interaction domain resided between amino acids 473–582 of HAP1. This region is represented as the TBP interacting region 2 in Fig. 5. The amino acid sequences of TBP interacting domains 1 and 2 show no obvious similarity (Fig. 5, bottom), suggesting that these are each unique interacting domains rather than redundant versions of the same domain.

### Intracellular co-localization of TBP and HAP1

**Figure 6:**
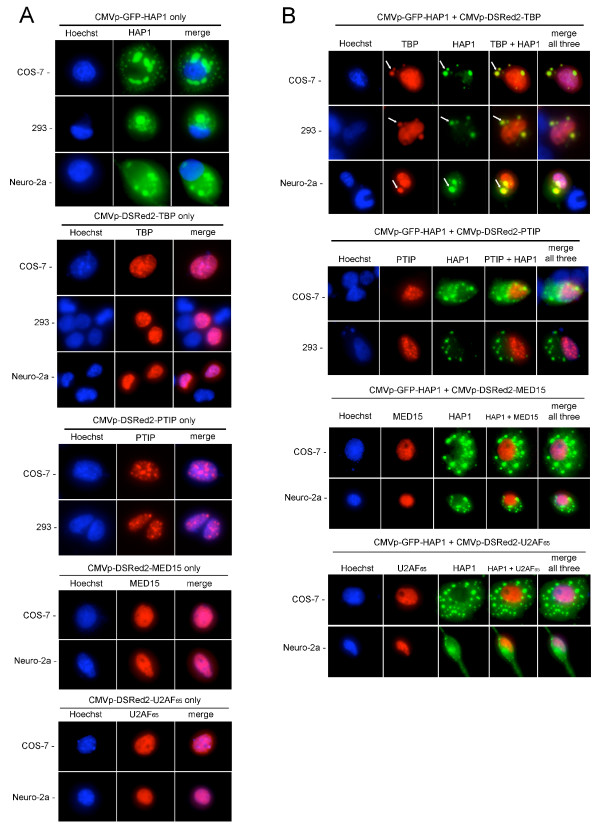
TBP and HAP1 co-localization assay in COS-7, 293, and Neuro-2a cells. (A) cells transfected with a single expression plasmid, as indicated at top. Forty-eight hours after transfection, cells were stained with Hoechst 33342 and fluorescent photomicrographs of the blue, red, and green channels were taken. Hoechst stain (blue) shows nuclei. CMVp-GFP-HAP1-A_7–598 _was exclusively cytoplasmic in all cells tested, where it assembled into strongly fluorescent STLBs. CMVp-DSRed2-TBP was exclusively nuclear with heterogenous subnuclear distributions when expressed alone. The negative controls, CMVp-DSRed2-PTIP, MED15, and U2AF_65 _(encoding nuclear proteins unrelated to TBP, see text), were also localized entirely to the nucleus when expressed alone. (B) co-expression of HAP1 with TBP, PTIP, MED15, or U2AF_65_. Co-expression of CMVp-GFP-HAP1 and CMVp-DSRed2-TBP shows that all HAP1 remained cytoplasmic, where it assembled into STLBs; however, TBP localization was altered. Thus, whereas much of the TBP still localized to the nucleus, a portion was sequestered into GFP-HAP1-STLBs. White arrows designate representative examples of extranuclear TBP in STLBs. As negative controls, DSRed2-PTIP, MED15, and U2AF_65 _were not detected in GFPHAP1-STLBs.

The TBP-HAP1 interaction was unexpected because, to our knowledge, following its synthesis in the cytoplasm, all TBP localizes to the nucleus [19]. Conversely, HAP1 is predominantly a cytoplasmic protein [34,42]. Nevertheless, the appearance of HAP1 in TBP-interaction screens using different libraries as well as the apparent specificity of interaction with the highly conserved TBP_CORE _region (Figs 2, 4) encouraged us to test whether we could detect colocalization of these two proteins in living cells. Expression vectors encoding TBP or HAP1-A fused to red or green fluorescent proteins, respectively, were constructed and transfected either individually (Fig. 6A) or in combination into COS-7, 293, or Neuro-2a cells (Fig. 6B). As negative controls, other nuclear proteins were fused to the red fluorescent protein and transfected either alone or in combination with GFP-HAP1. Specifically, we chose three representative proteins: MED15 (PCQAP), a component of the mediator [43,44], which is closely associated with TBP and the basal transcription machinery; PTIP [45], a DNA-binding transcription factor that associates with active chromatin; and U2AF_65 _(U2AF2), a component of the splicing machinery [46,47].

When either red fluorescently labeled TBP, MED15, PTIP, or U2AF_65 _were transfected alone, each of these proteins localized exclusively to the nucleus where each exhibited distinct heterogenous distributions (Fig. 6A). Conversely, fluorescently labeled HAP1 was exclusively cytoplasmic, where it assembled into STLBs [42] (Fig. 6A). In cells expressing both TBP and HAP1, however, a portion of the TBP co-localized with HAP1 in cytoplasmic STLBs (Fig. 6B). We did not detect any HAP1 redistributing to the nucleus with TBP. Co-expression of HAP1 with either MED15, PTIP, or U2AF_65 _did not result in any redistribution of these non-TBP nuclear proteins into STLBs (Fig. 6B). These results suggest that TBP can be sequestered in cytoplasmic STLBs *in vivo *via specific interactions with HAP1. Importantly, not all TBP was sequestered in STLBs (Fig. 6B). This suggests the association did not disrupt normal nuclear localization and function of TBP, but rather, was selective for only a subset of TBP in the cells.

**Figure 7:**
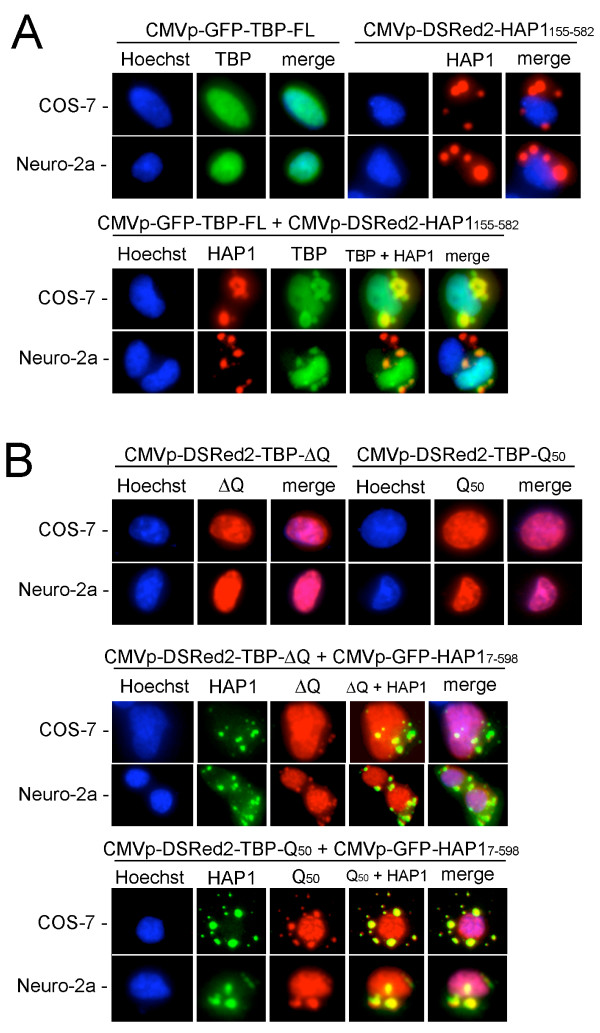
HAP1 and TBP-FL, -ΔQ, or -Q_50 _co-localization assays in COS-7 and Neuro-2a cells. (A) Top two rows show cells transfected with a single expression plasmid: CMVp-GFP-TBP-FL (at left) and CMVp-DSRed2-HAP1-B_155–582 _(at right). GFP-TBP was exclusively nuclear and RFP-HAP1 assembled into cytoplasmic STLBs. Bottom two rows show co-transfected cells containing GFP-TBP and RFP-HAP1. Upon co-expression with HAP1, a portion of GFP-TBP became localized to the cytoplasmic, RFP-HAP1-STLBs. (B) Top two rows show cells transfected with a single expression plasmid: CMVp-DSRed2-TBP-ΔQ (at left) and CMVp-DSRed2-TBP-Q_50 _(at right). Both RFP-TBP-ΔQ and RFP-TBP-Q_50 _proteins localized exclusively to the nucleus when expressed alone. Cells co-transfected with GFP-HAP1-A_7–598 _and either RFP-TBP-ΔQ (middle two rows) or RFP-TBP-Q_50 _(bottom two rows) showed altered localization of TBP away from the nucleus. RFP-TBP-ΔQ, which lacks the polyQ repeat, and RFP-TBP-Q_50_, which contains an expanded polyQ repeat, each were partially localized in the GFP-HAP1-STLBs.

HAP1-A has been shown to have a strong propensity toward forming STLBs in mammalian cells, whereas HAP1-B has been reported to have a more diffuse cytoplasmic expression pattern [36,48]. Our interaction data showed that TBP interacts with HAP1 in the region that is common to both HAP1 isoforms (Figs 4, 5). Moreover, our two-hybrid screens isolated a nearly full-length clone of HAP1-A from the oligo(dT)-primed library and a shorter clone from the random-primed library that extended five amino acids past the HAP1 common region into HAP1-B sequences. The nearly full-length HAP1-A clone, when fused to the DSRed2 fluorescent protein, formed strong STLBs and sequestered a fraction of co-expressed TBP protein in all mammalian cell types tested (Fig. 6). Interestingly, the short clone of HAP1-B (amino acids 155–582, including only five HAP1-B-specific amino acids) also assembled into STLBs and sequestered TBP protein (Fig. 7A). In combination with previous studies [36,48], these results suggest that the HAP1-A-specific C-terminal domain is not necessary for STLB assembly, but rather, the HAP1-B-specific C-terminus antagonizes STLB formation (see Discussion).

Although our two-component two-hybrid tests showed that the TBP polyQ region was not required for the TBP-HAP1 interaction (Fig. 2), the interaction of HAP1 with other polyQ proteins has been shown to be influenced by the length of polyQ repeats outside of their HAP1-binding domains [6,12,27,49]. The length of the polyQ region in vertebrate TBP proteins has been shown to be highly variable both between and within species [50-52]; polyQ expansion beyond 36 residues in human TBP causes SCA17 [6]. Therefore, we wished to test whether, in the context of a mammalian cell, we could detect quantitative differences in the amount of TBP that assembled into STLBs by altering the length of the polyQ repeat. Mutant versions of mouse TBP expression vectors were constructed that either replaced the normal 13-residue polyQ region with two glycines (TBP-ΔQ) or expanded the region nearly four-fold to contain 50 consecutive Q residues (TBP-Q_50_). When expressed alone, all three versions of full-length TBP (wild-type, ΔQ, and Q_50_) were exclusively nuclear (Figs 6A, 7B). When co-expressed with HAP1, all three full-length TBP proteins showed partial sequestration into cytoplasmic STLBs (Figs 6B, 7B).

**Figure 8:**
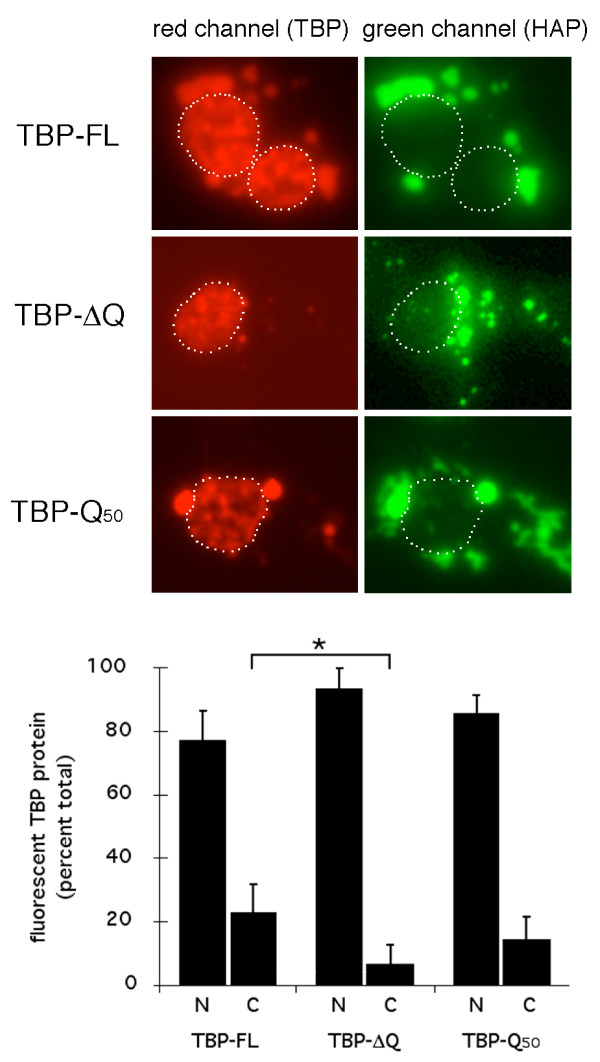
PolyQ dependence of relative nuclear and cytoplasmic TBP protein levels. (A) representative fluorescent photomicrographs used for quantitative analyses. Cells were transfected with CMVp-GFP-HAP1-A_7–598 _and either CMVp-DSRed2-TBP-FL, CMVp-DSRed2-TBP-ΔQ, or CMVp-DSRed2-TBP-Q_50_, as indicated at left. White dotted line delineates nuclei in each frame. (B) nuclear-cytoplasmic TBP distributions. Bars show average plus one standard deviation. Aserisks, cytoplasmic levels of TBP-ΔQ were significantly less than those of TBP-FL, P < 0.05. Cytoplasmic levels of TBP-FL and TBP-Q_50 _were not significantly different.

To quantify the proportion of TBP proteins assembling into STLBs, we co-transfected cells with GFP-HAP and either DSRed2-TBP-FL, DSRed2-TBP-ΔQ, or DSRed2-TBP-Q_50 _and measured nuclear and extra-nuclear TBP fluorescence (Fig. 8). Results showed that, whereas both wild type TBP and TBP-Q_50 _distributed similarly between the nucleus and cytoplasm, TBP-ΔQ exhibited significantly less association with cytoplasmic STLBs.

## Discussion

HAP1 is a predominantly cytoplasmic protein that has been implicated in contributing to various processes [42,53]. Since HAP1 has little similarity with other known proteins [33], much of what is known about its function has been discovered through identification of those proteins that interact with HAP1. Specific protein interaction sites on HAP1 have been mapped as follows (mouse HAP1-B amino acid numbering): amino acids 277–369 (HAP1_277–369_) binds with Htt; full-length HAP1-A binds to AR; HAP_277–444 _binds with dynactin P150^Glued^; HAP1_245–371 _binds with hepatocyte growth factor-regulated tyrosine kinase substrate protein; HAP1_1–312 _binds with interRho-GEF Kalirin-7; HAP1_219–519 _binds to GABA_A _receptor; HAP1_272–598 _binds to IP_3_1 receptor; and HAP1_247–446 _binds to NeuroD (ND) [27,33,42,53-57]. Here we show that a clone encoding amino acids 155–582 of HAP1 bound to the TBP_CORE _domain in yeast two-hybrid screens (Fig. 2). We also show that TBP co-immunoprecipitated with HAP1 in transient cotransfection assays (Fig. 3). Further, two regions within HAP1 were shown to independently interact with TBP-C. Specifically, the domains of HAP that interacted with TBP are found between amino acids 157–261 and between amino acids 473–582 (Figs. 4 and 5).

We are aware of only one study that has shown a role of HAP1 in transcriptional regulation. In that study, HAP1, in conjunction with Htt, was shown to function as a scaffold protein that mediates the interaction of ND with mixed-lineage kinase 2 in the cytoplasm, resulting in phosphorylation of ND and its subsequent translocation to the nucleus [57]. No evidence exists for cytoplasmic regulation of TBP activity, so we feel that it is unlikely that the TBP-HAP1 interaction might similarly regulate gene expression in HAP1-expressing cells. Rather, as detailed below, we favor a model based on the 'HAP1 protection hypothesis' [25], wherein HAP1 may help protect neuronal cells from TBP-mediated cytotoxicity.

Including this report, HAP1 has now been shown to bind specifically to three proteins associated with polyQ-dependent neuropathologies: Htt, AR, and TBP [4,5,27,53]. In all cases, recognition and binding of these proteins by HAP1 is dependent on unique sequences outside of their polyQ regions [4,27]. In other words, HAP1 does not recognize an expanded polyQ repeat per se, but rather, recognizes unique aspects of at least three specific proteins that have a propensity for causing polyQ-dependent neuropathology. Neruopathologies caused by Htt, AR, and TBP are associated with nuclear accumulation of protein aggregates, which are thought to induce cytopathic effects resulting in apoptotic neuron loss [1,3,20,53,58]. HAP1 is not associated with Htt aggregates in HD brains and HD neuropathology is not associated with brain regions having high HAP1 expression [5,28,34]. Thus, although Htt is expressed in many regions of the brain [59], regions that overlap with HAP1 expression are not vulnerable to neuropathology, leading to formulation of the 'HAP1 protection hypothesis' [25]. Similarly, although both bulbar and forebrain neurons express high levels of AR, polyQ-expanded AR aggregates and neuropathology in SBMA patients are not observed in the forebrain regions, which express high levels of HAP1 and exhibit STBs, but rather, are restricted to the HAP1/STB-deficient bulbar regions [25,27]. Overexpression of polyQ-expanded AR in HEp-2 cells induces apoptosis; however, co-overexpression of HAP1 suppresses apoptosis and, instead, causes formation of STLBs containing both proteins [27]. Thus, HAP1 binds to polyQ-expanded AR though specific interactions with regions outside the polyQ domain, it sequesters polyQ-expanded AR in STLBs, and it protects cells from polyQ-expanded AR-induced apoptosis [27], strongly supporting a protective role for HAP1.

Although it is not clear whether HAP1 might also protect neurons from TBP-mediated neuropathology, it is intriguing that, despite ubiquitous expression of TBP in all cells of the body, most regions of the brain exhibiting high levels of HAP1 and abundant STBs do not exhibit neuropathology in SCA17 patients [4-6,20,23,29]. One exception is in the striatum, which has both STBs [34] and SCA17-associated neuropathology [20,23]. However, detailed analyses show that, whereas HAP1/STBs are found in the medium and large neurons of the striatum [34], SCA17 is associated with loss of the small neurons [20,23].

In the current study, we show that HAP1 binds specifically to the TBP_CORE _and that HAP1 can sequester a subset of TBP into STLBs in co-transfected 293, COS-7, or Neuro-2a cells. Unlike the case for HAP binding to AR (see above), the HAP1-TBP interaction in yeast or mammalian cells did not require expansion of the polyQ region of TBP (Figs 2B, 4, 7, 8); however, removal of the polyQ region reduced assembly of TBP into STLBS (Figs 7, 8). Since the HAP1-binding domains of TBP, Htt, and AR are all disparate from the polyQ regions in each protein (see above), it is interesting that HAP1 binding by all three proteins should show polyQ-dependence. In transfected cells, HAP1/STLBs did not clear TBP from the nucleus (Fig. 6, 7, 8). Instead, they appeared to sequester a subset of TBP, while still allowing substantial TBP accumulation in the nucleus. It remains unclear whether the TBP protein that accumulated in the nucleus was equivalent to that sequestered in the STLBs (Figs 6, 7, 8). One possibility is that the polyQ region of each protein can influence the likelihood that that protein assumes a "state" that allows interaction with HAP1. For example, in the case of TBP, the subset of protein that is sequestered into HAP1 STLBs might be mis-folded, defective in nuclear localization, abnormally associated with other proteins, or otherwise defective, thus making this protein accessible for HAP1 binding. Further studies will be required to better understand the role of the TBP polyQ region in *in vivo *HAP1 association.

## Conclusion

HAP1 has previously been shown to interact specifically and away from the polyQ repeat region with Htt and AR proteins, the causative agents of HD and SBMA, respectively [27,53]. In the current study, we isolated HAP1 as a TBP_CORE_-interacting protein from unbiased two-hybrid screens. The interacting domains were mapped and the interactions were confirmed in co-immunoprecipitation assays. Sub-cellular co-localization studies indicated that HAP1-induced STLBs could sequester a subset of overexpressed TBP, but not other overexpressed nuclear proteins, away from the nucleus. Although the co-localization was modeled in an overexpression system, we suspect this may be a suitable model for a HAP1-TBP interaction. Thus, in normal cells, all TBP goes to the nucleus where it functions in transcription initiation and a HAP1-TBP interaction would not occur. Only in the case of a TBP expression anomaly would there be a possible physiological situation in which a HAP1-TBP interaction may be important to combat TBP-mediated cytotoxicity. In our co-localization system, the anomaly is strong overexpression of TBP in the transiently co-transfected cells; in SCA17 patients, anomalous expansion of the TBP polyQ repeat results in overaccumulation TBP, which assembles into nuclear aggregates and results in apoptotic neuronal loss [6]. Interestingly, neurons expressing HAP1 and possessing STBs appear to be refractory to nuclear aggregate formation and SCA17 cytopathology (see above). Thus, we posit that HAP1 plays a neuroprotective role in preventing nuclear aggregate formation in the presence of anomalous TBP accumulation.

## Methods

### Material availability

All renewable reagents generated for this study, including all plasmids, expression vectors, yeast two-hybrid libraries, and yeast strains, are available on request for unrestricted non-profit research use unless specifically regulated by other parties.

### Two-hybrid baits, libraries, transformations, and screens

TBP baits were generated by polymerase chain reaction (PCR) from a plasmid containing the predominant mouse (Mus musculus) somatic TBP cDNA [52,60]. Full-length TBP (TBP-FL) was amplified by PCR using TBP-N-start-*Sal *I primer and TBP-C-end primer; the TBP N terminus clone (TBP-N), which encoded amino acids 1–136, was amplified using TBP-N-start-*Sal *I primer and TBP-N-end primer; the TBP_CORE _clone (TBP-C), which encoded amino acids 134–316, was amplified using TBP-C-start primer and TBP-C-end primer (Table 1). TBP-FL and TBP-N were cut with *Sal *I and *Not *I and inserted into the pDBLeu bait vector (Gibco BRL ProQuest Two-Hybrid System). TBP-C was digested with *Nco *I and *Not *I and inserted into pDBLeu. TBP-ΔQ was constructed using primers TBP-N-start-*Sal *I and TBP-ΔQ and inserted into a plasmid containing the TBP-N. The TBP-ΔQ N region was then sub-cloned into TBP-FL. The resulting clone inserts a Gly-Gly linker in place of the polyQ repeat region (amino acids 55–70 of the mouse TBP-FL protein). TBP-ΔQ was digested with *Sal *I and *Not *I and was inserted into pDBLeu. Truncated TBP_CORE _clones were constructed as described [61] and were inserted between *Sal *I and *Not *I in the pDBLeu plasmid.

**Table 1:** Oligonucleotide sequences

name	sequence^1^
GFP-N-start	5'-tat*gcggccgcgtcgac*c**atggtgagcaagggcgaggagct**-3'
GFP-C-end	5'-tat*gcggccgc*a*gtcga****c*ttgtacagctcgtccatgcc**-3'
HAP1-157-forward	5'-tat*gtcgac***agaacgggacctgaacacagc**-3'
HAP1-238-forward	5'-tat*gtcg****ac*acagagggatcaagaccagca**-3'
HAP1-300-forward	5'-tat*gtcgac***gatgctcattctggaatgtgtg**-3'
HAP1-380-forward	5'-tat*gtcgac***ctcctacatgcaggattatggg**-3'
HAP1-473-forward	5'-tat*gtcgac***agatctcaagccacctgaagat**-3'
HAP1-261-reverse	5'-tat*gcggc****cgc*tgtctcagccttagggtgt**-3'
HAP1-306-reverse	5'-tat*gcggccg****c*acacattccagaatgagcatc**-3'
HAP1-386-reverse	5'-tat*gcggccg****c*ccataatcctgcatgtaggag**-3'
HAP1-478-reverse	5'-tat*gcggccgc***tcttcaggtggcttgagatctt**-3'
HAP1-582-reverse	5'-tat*gcggccgc***gttggggagtcttttgggacca**-3'
TBP-N-start-*Sal *I	5'-atc*gtcgac*t**atggaccagaacaacagccttcca**-3'
TBP-N-start-*Bgl *II	5'-tat*agatct***atggaccagaacaacagccttc**-3'
TBP-N-end	5'-ata*gcggccgc*ttaa**gagctctcagaagctggtgtggca**-3'
TBP-C-start	5'-tatggatcca*ccatgg*accagagc**tctggaattgtaccgcagcttca**-3'
TBP-C-end	5'-gcta*gcggccgc*ccaagtagcagcacagagc-3'
TBP-Q_50_	5'-ata***ctgcag*ttgctactgc**ctgttgctgctgttgctgctgctgttgctgttgctgctgttgctgttgctgctgttgttgctgctgctgttgctgctgctgctgttgttgctgctgttgctgctgttgctg**ctgctgttgttgctgctgctgttgctgttgctgctgctgtctttgttgctcttccaaaatagagagact**-3'
TBP-ΔQ	5'-atgca*ctgcagtcgcga*cagctccgcc**ctcttccaaaatagagagact**-3'
U2AF-N-start	5'-tat*gtcgac*c**atgtcggacttcgacgagttcgag**-3'
U2AF-C-end	5'-tat*gcggccgc***cggtggtaagaatcagggtca**-3'
linker (top strand)	5'-aattc*gcggccgcgtcgac*-3'
linker (bottom strand)	5'-phos-*gtcgacgcggccgc*g-3'

^1^Endogenous protein-coding sequences are in bold and engineered restriction sites are in italics.

Oligo(dT)-primed cDNA prey libraries were constructed and inserted into the pPC86 vector (Gibco BRL ProQuest) as follows. Total RNA was extracted and CsCl-purified [22] from embryonic day 10.5 (E10.5) wild-type C57Bl/6J whole pregnant uteri or placentas. In each case, either whole pregnant uteri or placentas were obtained from four pregnant dams to generate a pool of RNA. Poly(A+) mRNA from these samples was purified using Oligo(dT)_25 _Dynabeads (Dynal Biotech ASA) following the manufacturer's protocols. Oligo(dT)-primed libraries were constructed from ~3 μg of poly(A+) RNA; cDNA inserts for each library had an average size of >1 kb (Fig. 1B). The whole pregnant uteri and placental libraries contained ~2.1 × 10^6 ^and ~2.5 × 10^6 ^independent recombinants, respectively; each with ~95% bearing inserts (Fig. 1B). To access more 5' regions of prey cDNAs, a random hexamer-primed library was also constructed using 3 μg each of whole pregnant uteri and placental poly(A+) RNA. Construction of random-primed cDNAs was performed using a standard oligo(dT)-primed cDNA synthesis kit (Stratagene), with the following adaptations. First, instead of an oligo(dT) primer, random hexamer oligonucleotides were used to prime the first-strand cDNA. Second, a linker containing a 5' *Eco *RI overhang and a blunt 3' end was constructed by annealing the 5' and 3' linker oligos (Table 1) and ligating this to the blunt-ended double-strand cDNA product. Both the 5' and 3' ends of the cDNA had *Eco *RI overhangs (Table 1), which allowed direct insertion of the library product into an *Eco *RI-digested vector. Two versions of the random-primed library were constructed and subsequently pooled. The first consisted of non-amplified cDNA that was inserted into the *Eco *RI site of pPC86. This library contained ~6 × 10^5 ^primary transformants with ~60% bearing inserts. The second library was constructed from random-primed cDNA that had been amplified by PCR using primers that annealed to the linker sequences located at the 5' and 3' ends of the final cDNA library product. PCR-amplified cDNA was digested with *Not *I and inserted into the *Not *I site of pPC86. This library contained ~1.8 × 10^6 ^independent recombinants with ~90% bearing inserts. After pooling, the combined library consisted of ~2.4 × 10^6 ^cDNA clones and had an average insert size of ~1.0 kb with ~90% bearing inserts (Fig. 1B).

All interactions were tested in Saccharomyces cerevisiae strain MaV203 (Gibco BRL), which contained the HIS3, URA3, and LacZ reporters, each under the control of a different promoter. For library transformations, a culture of MaV203 that contained the TBP bait construct was grown at 30°C for 48 hours in liquid synthetic complete medium (SC) lacking leucine (SC-L) (QBIOgene). This culture was used to seed 300 ml of 2× yeast extract/peptone/adenine/dextrose (YPAD) at 5 × 10^6 ^yeast/ml, which was determined by counting on a hemacytometer. The culture was grown at 30°C for ~5 hours to a density of 2 × 10^7 ^yeast/ml. Yeast were collected by centrifugation, washed once with water and once with 100 mM LiAc, and transformed with 14.4 ml 50% PEG (average m.w. 3350), 2.16 ml 1.0 M LiAc, 0.3 ml 10 mg/ml sheared denatured salmon sperm DNA, and 120 μg of the cDNA library in pPC86. Single- or two-component transformations of bait and/or prey plasmids into yeast used a standard PEG/LiAc protocol [62].

Two-hybrid screens were performed on SC medium lacking leucine, tryptophan, histidine, and uracil (SC-L-W-H-U). Both two-hybrid screens used TBP-FL as bait to screen either a combination of 50% whole pregnant uteri and 50% placental (screen 1) or random-primed (screen 2) cDNA prey libraries. Primary transformants were transferred onto a new SC-L-W-H-U plate and clones that grew well on these plates were transferred to either a SC-L-W-H + 80 mM 3-AT plate or were grown on SC-L-W and tested for LacZ expression following the X-Gal Assay protocol detailed in the ProQuest Two-Hybrid system manual (Gibco BRL cat. series 10835). Prey plasmids from clones that grew under all selection conditions and scored positive for LacZ expression (turned blue) were isolated from yeast by glass bead lysis [63]. Recovered plasmids were transformed into bacteria, clones were selected, and inserts were sequenced to determine the cDNA identity. Isolated prey plasmids were re-transformed into MaV203 with the bait or with the empty pDBLeu plasmid (auto-activation test) and grown on SC-L-W-H + 75 mM 3-AT, and SC-L-W-H-U plates to verify the interaction.

To identify the domain of TBP that interacted with HAP1, TBP bait constructs in combination with the empty pPC86 prey plasmid (auto-activation test) or with either the HAP1 random-primed or the HAP1 oligo(dT)-primed clone were co-transformed into MaV203 and plated onto SC-L-W. Individual colonies that grew on SC-L-W plates (containing both the bait and prey plasmid) were suspended in water and streaked onto SC-L-W, SC-L-W-H + 75 mM 3-AT, and SC-L-W-H-U plates to verify the interaction.

### Interaction domain mapping

For generating a HAP1 deletion mutant series, a random-primed yeast two hybrid prey plasmid isolated as a TBP interactor, encoding amino acids 155–582 of the mouse HAP1-B cDNA, was used as the template for PCR amplification. PCR primer sets for amplification of each truncated clone are indicated in Table 1. PCR products were digested with *Sal *I and *Not *I and were inserted into the pPC86 prey vector.

Three plasmid clones of each truncated HAP1 construct were isolated and separately co-transformed into yeast with pDBLeu-mTBP-N, pDBLeu-mTBP-C, or empty pDBLeu bait plasmid. Yeast were plated onto SC-L-W agar plates and grown at 30°C. Resultant colonies were suspended in water and streaked onto SC-L-W, SC-L-W-H + 75 mM 3AT, and SC-L-W-H-U to test each protein interaction pair.

TBP-ΔQ and truncated TBP-C clones were co-transformed into yeast with pPC86-mHAP1-A_7-598 _or the empty pPC86 prey plasmid. Yeast were plated onto SC-L-W agar plates and grown at 30°C. Two resultant colonies for each yeast co-transfection were suspended in water and streaked onto SC-L-W, SC-L-W-H + 75 mM 3AT, and SC-L-W-H-U to test each protein interaction pair.

### Co-immunoprecipitation assays

A HAP1 cDNA encoding amino acids 155–582 of the mouse HAP1-B protein was inserted into the *Eco *RI site of pCMV-MYC (BD Biosciences). The TBP-FL cDNA was inserted between the *Sal *I and *Not *I sites in both pCMV-HA (BD Biosciences) and pFLAG-CMV-2^SN^, which is a modified version of pFLAG-CMV-2 (Sigma). pFLAG-CMV-2^SN ^contained a modified polylinker, and was constructed by inserting a linker oligonucleotide containing internal *Sal *I and *Not *I sites and a 5' *Hind *III and 3' *Bam *HI into *Hind *III/*Bam *HI-digested pFLAG-CMV-2. A green fluorescent protein- (GFP-) tagged TBP construct, pFLAG-CMV-GFP-TBP-FL, was constructed by PCR amplification of EGFP with primers GFP-N-start and GFP-C-end (Table 1). PCR-amplified EGFP was digested with *Sal *I and inserted into the *Sal *I site upstream of TBP-FL in pFLAG-CMV-2^SN^.

Line 293 cells were transfected and lysates were prepared as described previously [61]. Lysates were pre-adsorbed with 10 μl of non-specific rabbit polyclonal antibody and 20 μl of Protein G plus Protein A agarose (Calbiochem) for 2 hours, rotating at 4°C, after which the agarose was pelleted and 0.5 ml of supernatant was transferred to a new tube containing 12.5 μl of either anti-TBP N_C _rabbit polyclonal antibody [61] or a non-specific control antibody and rotated at 4°C for 16 hours, followed by one hour with 30 μl of Protein G plus/Protein A agarose added. Beads were washed once with lysis buffer, four times with wash buffer (50 mM Tris-HCl, pH 7.4, 300 mM NaCl, 2% TX-100, 10% glycerol, and 1 mM PMSF), and a final time with wash buffer that contained 100 mM NaCl. Proteins bound to agarose beads were released by boiling for 5 minutes in 1× SDS-PAGE sample buffer, separated by polyacrylamide gel electrophoresis, and analyzed by Western blotting with the antibodies indicated in figure legends.

COS-7 cells at 40–50% confluence on 10 cm dishes were transfected and lysates were prepared as described previously [61], with the following modifications. Cells were lysed in 750 μl of lysis buffer (50 mM Tris, pH 7.4, 150 mM NaCl, 1% TX-100, 0.1% SDS, 0.5% deoxycholic acid, 10% glycerol, 1 mM EDTA, 1 mM PMSF, 5 μg/ml leupeptin-pepstatinaprotinin, and 0.1 mM Na_3_V0_4_). After lysis, cells were brought to a final volume of 1.5 ml with 150 mM NaCl wash buffer (50 mM Tris, pH 7.4, 150 mM NaCl, 1% TX-100, 10% glycerol, 1 mM EDTA, 1 mM PMSF, 0.5 μg/ml leupeptin-pepstatin-aprotinin, and 0.1 mM Na_3_V0_4_). Lysates were pre-adsorbed with 2 μg of non-specific mouse monoclonal antibody and 20 μl of Protein G plus Protein A agarose (Calbiochem) for 2 hours, rotating at 4°C, after which the agarose was pelleted and 1.4 ml of supernatant was transferred to a new tube containing 5 μg of either anti-FLAG M2 monoclonal antibody (Sigma) or a non-specific control antibody and rotated at 4°C for 16 hours, followed by one hour with 40 μl of Protein G plus/Protein A agarose added. Beads were washed once with 150 mM NaCl wash buffer, three times with 300 mM NaCl wash buffer (50 mM Tris-HCl, pH 7.4, 300 mM NaCl, 2% TX-100, 10% glycerol, 0.5 μg/ml leupeptin-pepstatin-aprotinin, and 1 mM PMSF), and a final time with 150 mM NaCl wash buffer. Proteins bound to agarose beads were released by boiling for 5 minutes in 1× SDSPAGE sample buffer, separated by polyacrylamide gel electrophoresis, and analyzed by Western blotting with the antibodies indicated in the figure legend.

### In vivo co-localization assays

For co-localization assays, a full-length cDNA of mouse TBP was PCR-amplified with TBPN-start-*Bgl *II and TBP-C-end primers (Table 1), and inserted between the *Bgl *II and *Kpn *I sites in the pDsRed2-C1 plasmid (BD Biosciences). TBP-ΔQ was inserted between the *Sal *I and *Not *I sites in pDsRed2-C1^SN^, which is a modified version of pDsRed2-C1. pDsRed2-C1^SN ^was constructed by inserting a linker oligonucleotide containing internal *Sal *I and *Not *I sites and 5'*Eco *RI and 3' *Bam *HI overhangs into *Eco *RI/*Bam *HI-digested pDsRed2-C1. TBP-Q_50 _was constructed using primers TBP-N-start-*Sal *I and TBP-Q_50 _and inserted into a plasmid containing the TBP-N-terminus. The TBP-N-terminal clone containing a polyQ-repeat region expanded from 13- to 50-Q residues was then sub-cloned into TBP-FL, which was inserted into pDsRed2-C1^SN ^for use in co-localization assays.

As controls, three additional cDNAs were inserted into pDSRed2-Cl (MED15) or pDSRed2-C1^SN ^(PTIP and U2AF_65_). Clones were as follows: amino-acids 429–1056 of the nuclear protein PTIP [45]; the full-length mouse MED15 open reading frame (ATCC, catalog no. 10699004; NCBI accession number BC054779); and full-length mouse U2AF_65 _(NCBI accession number BC106134) [46,47], which was isolated by RT-PCR and subsequent PCR amplification of mouse C57Bl/6J cDNA with primers U2AF-N-start and U2AF-C-end (Table 1).

A GFP-tagged HAP1 protein was constructed by PCR amplification of EGFP with primers GFP-N-start and GFP-C-end (Table 1). The EGFP PCR product was digested with *Sal *I and inserted into the *Sal *I site upstream of HAP1-A_7–598 _in the pCMV-MYC plasmid. This resultant construct was termed pCMV-MYC-GFP-HAP1-A_7–598_. A red fluorescent protein- (RFP-) tagged HAP1 protein was also constructed by insertion of HAP1-B_155–582 _into the Sal I site in pDSRed2-C1.

Cell transfection conditions for 293, COS-7, and Neuro-2a cells were similar to those performed for co-immunoprecipitation assays except that 0.5 μg of each plasmid was used for each transfection. In single plasmid transfections, total DNA was brought to 1 μg with pBluescript II KS^+ ^plasmid (Stratagene). Forty-eight hours after transfection, cells were stained with Hoechst 33342 (Sigma) at 1.0 μg/ml in PBS for 15 min, rinsed once, given fresh complete medium, and photographed on a Nikon Eclipse TE300 inverted fluorescent microscope.

### Quantitation of relative nuclear and extranuclear TBP

COS-7 cells were transfected as above with the fluorescent fusion proteins indicated in figure legend. Forty-eight hours after transfection, cells were stained with Hoechst and digital fluorescent photomicrographs of cells expressing both HAP1 and TBP were taken (ten or more co-expressing cells per experimental condition were chosen arbitrarily). Hoechst and GFP-HAP1 fluorescence were used to delineate nuclear and cytoplasmic regions, as indicated by white dotted lines in Fig 8A. Because adherent cells have little cytoplasm over- or under-lying the nucleus, most cells gave clear spatial separation of GFP-HAP1 STLB fluorescence and nuclear Hoechst fluorescence; however, a few cells did have evidence of STLBs over- or under-lying the nucleus, and these were excluded from the analysis.

Digital photomicrographs of individual cells were used to estimate relative nuclear and extranuclear TBP levels. Total area, nuclear area, and extranuclear area were analyzed using the "Image Histogram" tool for the red (DSRed2-TBP expression) channel within Photoshop 7.0 (Adobe). The pixel-count per intensity level curve was integrated to provide a quantitative estimate of the total amount of red fluorescence in each compartment. Because this integration takes into account the fluorescence intensity in each pixel, it converts the two-dimensional fluorescent photomicrograph into a quantitative representation of all fluorescence in the three-dimensional space within each cellular region, and thus provides an estimate of relative fluorescent protein concentrations.

## Competing interests

The author(s) declares that there are no competing interests.

## Authors' contributions

EES designed and directed the project and helped prepare the manuscript. JRP performed screens and experiments and helped prepare the manuscript. All authors have read and approved the final manuscript.
